# QT Interval Prolongation in Chronic Liver Disease: A Cross-Sectional Study

**DOI:** 10.7759/cureus.92804

**Published:** 2025-09-20

**Authors:** Thithiksha Venkata Harischandra, Ali Zulqurnain, Ali Irshad, Mavish Arif, Asma Abdul Razzak, Muhammad Tayyab, Adeel Ur Rehman

**Affiliations:** 1 Internal Medicine, Stanley Medical College, Chennai, IND; 2 General Medicine and Elderly Care, Warrington Hospital, Warrington, GBR; 3 Internal Medicine, Peshawar General Hospital, Peshawar, PAK; 4 Gastroenterology and Hepatology, Jinnah Medical and Dental College (JMDC) Karachi, Karachi, PAK; 5 Cardiology, Azra Naheed Medical College, Lahore, PAK; 6 Neurosurgery, Punjab Institute of Neurosciences, Lahore, PAK

**Keywords:** arrhythmias, chronic, electrocardiographic, liver fibrosis, patient

## Abstract

Background: Chronic liver disease (CLD) is a leading cause of morbidity and mortality worldwide, and its association with cardiovascular complications, particularly QT interval prolongation and arrhythmias, remains underexplored.

Objective: This study aimed to determine the prevalence of QT prolongation and cardiac rhythm disturbances in patients with CLD and to explore contributing factors, including liver disease severity, electrolyte abnormalities, and diuretic use.

Methods: A cross-sectional study was conducted at Warrington Hospital, Warrington, UK, from June 2024 to June 2025. A total of 155 consecutive patients suffering from CLD were added to the study. Demographic, clinical, and laboratory data were collected, and all participants underwent a 12-lead electrocardiogram (ECG) to assess QT interval.

Results: A total of 155 patients with CLD were enrolled in the study, with a mean age of 56.3 ± 12.1 years. Out of these, 60% (n = 93) were male, and 40% (n = 62) were female. Fifty-nine (38%) of patients had prolonged QT intervals, with 29 (45%) of those with cirrhosis showing QT prolongation compared to 30 (19%) in non-cirrhotic patients. Electrolyte imbalances, including hypokalemia (54, 35%) and hypomagnesemia (42, 27%), were more prevalent in patients with prolonged QTc intervals. Arrhythmias were detected in 70 (45%) of patients, with atrial fibrillation being the most common, occurring in 37 (24%). The presence of QT prolongation was significantly associated with an increased incidence of arrhythmias (58% vs. 30%, p = 0.002). Diuretic use was strongly associated with both QT prolongation (p = 0.005) and arrhythmias (p = 0.02). The mortality rate in the study cohort was nine (6%), with higher mortality observed in patients with both QT prolongation and arrhythmias (three, 10%).

Conclusion: QT interval prolongation and cardiac rhythm disturbances are highly prevalent in patients with CLD, especially those with cirrhosis and advanced disease. Electrolyte abnormalities and diuretic use were major contributors to these abnormalities.

## Introduction

Chronic liver disease (CLD) is a significant global health concern, encompassing a wide range of hepatic disorders, including cirrhosis and hepatitis. The progression of these disorders increases the risk of systemic complications, most notably the development of cardiac arrhythmia [1]. QT interval prolongation is a well-known electrocardiographic phenomenon among these arrhythmias and is directly correlated with an elevated risk of arrhythmic events leading to morbidity and even death (i.e., torsades de pointes and ventricular fibrillation) [2]. Although it is clear that the connection between QT prolongation and arrhythmias is well documented in a range of disorders, whether its effect is as pronounced in patients with CLD is a question of immense clinical interest [3]. Cirrhosis represents the terminal stage of chronic liver disease, characterized by portal hypertension and the formation of regenerative nodules encased in fibrous tissue, making it a major health burden in both developed and developing countries [4]. Liver cirrhosis is ranked as the 12th leading cause of death in the world, and it is a major cause of death because more than 27,000 people die each year, and over 421,000 are admitted to hospitals [5]. A close indicator of the electrophysiologic cardiac status is the QT interval, or the duration during which the ventricles repolarize following every heartbeat. In CLD, there are a number of pathophysiological causes of QT prolongation. These involve electrolyte imbalance (hypokalemia, hypomagnesemia, and hyponatremia), dysfunction of the autonomic nervous system, and the side effects of the pharmacological agents that are regularly prescribed in the management of CLD [6]. This can further compromise the metabolism of medication as well, given that the hepatic dysfunction is caused by the presence of cirrhosis and other advanced degenerative liver diseases, which further contribute to these disturbances and risks of developing arrhythmias [7].

CLD patients have a special clinical problem concerning arrhythmias. Atrial fibrillation, ventricular tachycardia, and sinus node dysfunction are the most frequent types of arrhythmia among patients of this population. Not only does the appearance of arrhythmias aggravate the treatment of liver disease, but it also leads to high morbidity and mortality [8]. Conditions that predispose patients to arrhythmic actions include hepatic encephalopathy, portal hypertension, and the presence of systemic inflammation related to the patient having liver disease. Notably, patients with severe liver disease risk of having arrhythmias than those with earlier stages, and some, including those with cirrhosis, are affected based on hemodynamic and metabolic alterations that come with this phase of liver malfunction [9]. Despite QT interval prolongation and arrhythmias being known complications of patients with CLD, the exact mechanisms that establish a link between these two complications are still not fully determined [10]. These abnormalities in the cardiac structure are caused by a variety of factors, such as disrupted hepatic drug metabolism, electrolyte disturbances, and the systemic inflammatory response present in the disease of the liver [11]. Moreover, the treatment of arrhythmias in CLD patients is also burdened by the fact that the choice of pharmacological tools is very limited, and the vast majority of them are either contraindicated or should be used with caution in patients with liver failure [12].

Objective

This study aimed to determine the prevalence of QT prolongation and cardiac rhythm disturbances in patients with CLD and to explore contributing factors, including liver disease severity, electrolyte abnormalities, and diuretic use.

## Materials and methods

This cross-sectional study was conducted at Warrington Hospital, Warrington, UK, from June 2024 to June 2025. A total of 155 patients diagnosed with chronic liver disease were included in the study. Patients were selected using a non-probability consecutive sampling method, whereby all eligible patients were enrolled. The sample size was determined based on statistical power calculations, considering the expected prevalence of QT prolongation and arrhythmias in this population. The sample size was calculated using the single-proportion formula, assuming a 38% prevalence of QTc prolongation, 95% confidence level, and 8% margin of error, giving a minimum of 142 patients. To improve precision and allow subgroup analysis, a total of 155 patients were enrolled. Data were collected after the approval of the ethical committee of the hospital. Patients aged 18 to 75 years, diagnosed with chronic liver disease, including hepatitis and cirrhosis, confirmed through clinical, biochemical, and imaging findings, were eligible for inclusion in the study. Patients with a history of known congenital arrhythmias or structural heart disease, experiencing severe acute liver failure or life-threatening complications of liver disease, active infections, or severe electrolyte imbalances at the time of enrollment were excluded from the study. Pregnant or lactating women were also excluded, as well as patients who were taking medications known to significantly alter QT intervals or arrhythmia risk, such as certain anti-arrhythmics, chemotherapy agents, or immunosuppressants.

Data collection

Demographic and clinical data were collected through a combination of structured patient interviews and review of medical records. The patient's age, gender, comorbid conditions (such as hypertension and diabetes), etiology of liver disease, liver disease severity (assessed using the Child-Pugh [13] or Model for End-Stage Liver Disease (MELD) score [14]), and medication history were recorded. Blood samples were taken for laboratory testing, including liver function tests (LFTs), renal function tests, and electrolyte levels (potassium, magnesium, calcium).

Electrocardiogram (ECG) measurement

All patients underwent a 12-lead electrocardiogram (ECG) to assess the QT interval. QT interval was manually measured on the ECG recording, and the lead with the longest QT interval was used (which is often lead II). We used the Bazett formula (QTc = QT / square root of (RR)) to calculate the corrected QT interval, and a QTc of more than 450 ms in men and 470 ms in women was determined to be prolonged. Those patients who experienced a long QTc interval were also examined to determine whether they had arrhythmia or not. ECG morphology helped to estimate important arrhythmias such as atrial fibrillation, arrhythmia in the ventricle, and other rhythm disorders. Blood samples were taken, and the same samples were used to perform general tests in the laboratory, such as liver functional tests (e.g., alanine aminotransferase (ALT), aspartate aminotransferase (AST), bilirubin), kidney tests (e.g., creatinine, blood urea nitrogen), as well as electrolyte testing (e.g., potassium, magnesium, calcium). Arrhythmias were identified based on the electrocardiogram findings. The primary focus was on atrial arrhythmias, such as atrial fibrillation and atrial flutter, and ventricular arrhythmias, including ventricular tachycardia and torsades de pointes. Sinus arrhythmia, bradycardia, and other significant rhythm disturbances were also documented.

Statistical analysis

The collected data were analyzed using SPSS version 26.0 (IBM Corp., Armonk, NY, USA). Descriptive statistics were used to summarize the demographic characteristics and clinical features of the study participants. Categorical variables were presented as frequencies and percentages, while continuous variables were expressed as means ± standard deviation (SD). Associations between QTc prolongation, electrolyte imbalances, diuretic use, and arrhythmias were assessed with χ² and t-tests, with odds ratios (OR) and 95% confidence intervals reported where appropriate. A p-value of ≤0.05 was considered statistically significant.

## Results

A total of 155 patients with CLD were enrolled in the study, with a mean age of 56.3 ± 12.1 years. Out of these, 60% (n = 93) were male, and 40% (n = 62) were female. Hepatitis C was the most frequent etiology (45%), followed by alcoholic liver disease (30%) and nonalcoholic fatty liver disease (NAFLD) (25%). Cirrhosis was present in 70% of cases (n = 108), while 30% (n = 47) had non-cirrhotic disease; among cirrhotics, 30% were Child-Pugh A, 43% Child-Pugh B, and 27% Child-Pugh C, indicating a large proportion with advanced liver dysfunction. Electrolyte disturbances were frequent, with hypokalemia in 35% and hypomagnesemia in 27% of patients. Prolonged QTc intervals were observed in 38% of the cohort, affecting 22% of men and 16% of women, with a higher prevalence among cirrhotic patients (45%) compared to non-cirrhotic patients (19%) (Table 1).

**Table 1:** Demographic and Clinical Characteristics of Study Participants CLD: Chronic liver disease

Characteristic	Value
Age (years), mean ± SD	56.3 ± 12.1
Gender, n (%)	
Male	93 (60)
Female	62 (40)
Etiology of Liver Disease, n (%)	
Hepatitis C	70 (45)
Alcoholic Liver Disease	46 (30)
Non-Alcoholic Fatty Liver Disease (NAFLD)	39 (25)
Cirrhosis, n (%)	108 (70)
Non-Cirrhotic CLD, n (%)	47 (30)
Child-Pugh Class (Cirrhosis only, n=108), n (%)	
Class A (5–6)	32 (30)
Class B (7–9)	46 (43)
Class C (10–15)	30 (27)
Electrolyte Imbalances, n (%)	
Hypokalemia	54 (35)
Hypomagnesemia	42 (27)

Out of the patients with prolonged QTc intervals (n = 59), 66% (n = 39) had hypokalemia and 54% (n = 32) had hypomagnesemia, compared to only 16% (n = 15) and 10% (n = 10) of patients with normal QT intervals (n = 96), respectively. Serum potassium and magnesium levels were also significantly lower in those with prolonged QTc, with mean values of 3.4 ± 0.6 mEq/L for potassium and 1.4 ± 0.4 mg/dL for magnesium, compared to 4.0 ± 0.5 mEq/L and 1.8 ± 0.3 mg/dL in the normal QT group. These associations were statistically significant (p = 0.004 for hypokalemia, 0.01 for hypomagnesemia, 0.003 for potassium, and 0.01 for magnesium) (Table 2).

**Table 2:** Electrolyte Imbalances and QT Prolongation

Electrolyte Imbalance	Normal QTc Interval (n = 96)	Prolonged QTc Interval (n = 59)	p-value	t-value	Chi-square value
Hypokalemia	15 (16%)	39 (66%)	0.004	-	18.45
Hypomagnesemia	10 (10%)	32 (54%)	0.01	-	16.67
Mean Serum Potassium (mEq/L)	4.0 ± 0.5	3.4 ± 0.6	0.003	3.42	-
Mean Serum Magnesium (mg/dL)	1.8 ± 0.3	1.4 ± 0.4	0.01	2.94	-

**Figure 1:**
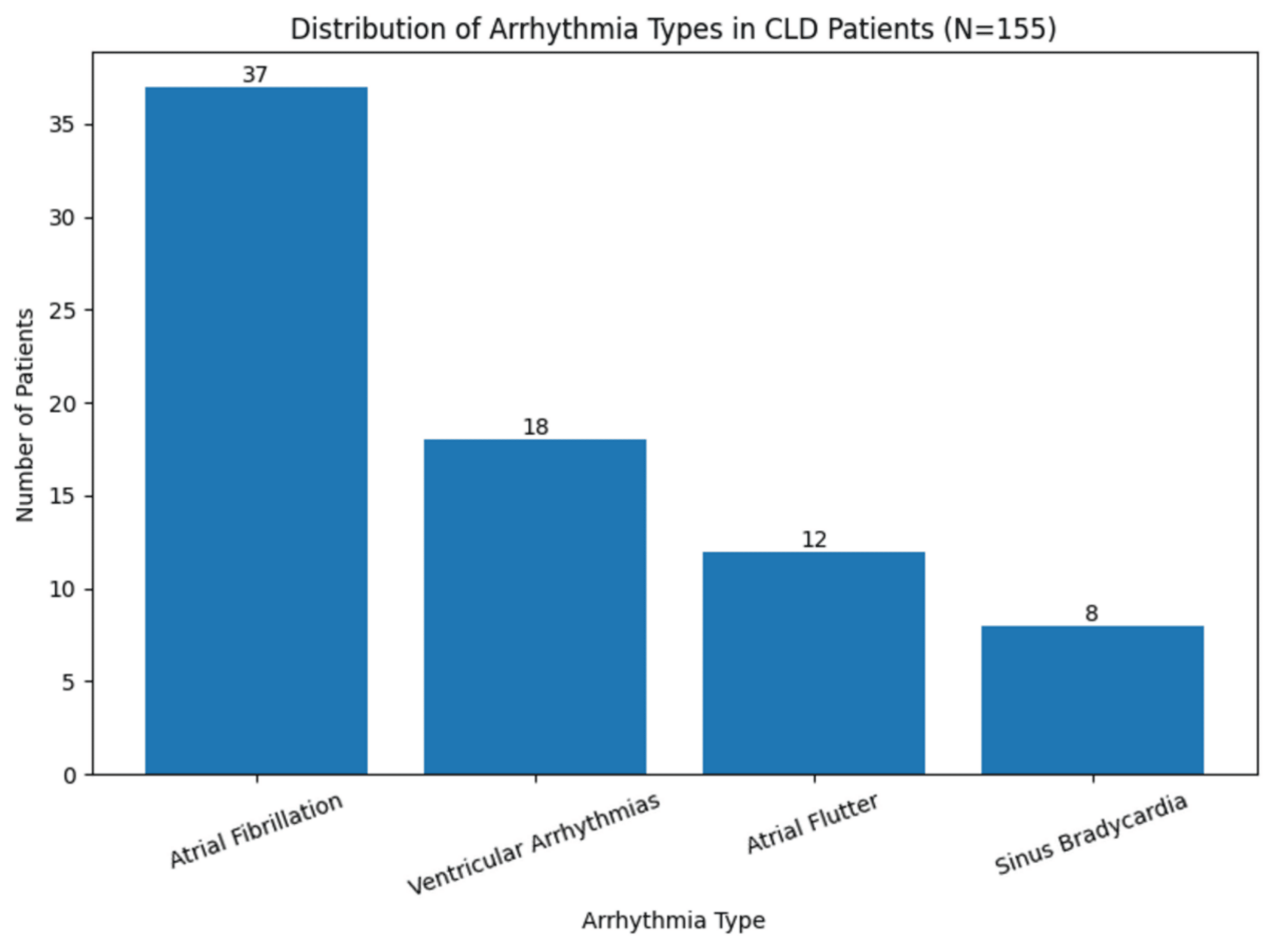
Distribution of Arrhythmia Types in Chronic Liver Disease (CLD) Patients (N=155)

Arrhythmias were identified in 45% of patients, with atrial fibrillation being the most common rhythm disturbance (24%). Stratification by liver disease severity revealed a clear gradient: 31% in Child-Pugh A, 43% in Child-Pugh B, and 60% in Child-Pugh C, compared with 47% in non-cirrhotic patients (Table 3, Figure 1).

**Table 3:** Prevalence of Arrhythmias CLD: Chronic liver disease

Characteristic	Value, n (%)
Any Arrhythmia	70 (45)
Atrial Fibrillation	37 (24)
Ventricular Arrhythmias	18 (12)
Atrial Flutter	12 (8)
Sinus Bradycardia	8 (5)
Arrhythmias by Liver Disease Severity	
Non-Cirrhotic CLD (n = 47)	22 (47)
Child-Pugh A (n = 32)	10 (31)
Child-Pugh B (n = 46)	20 (43)
Child-Pugh C (n = 30)	18 (60)

Among patients with prolonged QTc intervals, 58% (n = 41) had arrhythmias, compared to only 30% (n = 18) of those without QT prolongation. In contrast, among patients with normal QTc intervals, 42% (n = 29) had arrhythmias, compared to 70% (n = 67) of those without arrhythmias (Table 4).

**Table 4:** Association Between QT Prolongation and Arrhythmias

QT Prolongation Status	Arrhythmia Present (n = 70)	No Arrhythmia (n = 85)	p-value	Chi-square value
Prolonged QTc Interval	41 (58%)	18 (30%)	0.002	9.48
Normal QTc Interval	29 (42%)	67 (70%)		

Among the 121 patients receiving diuretics, 51 (42%) developed QT prolongation while 70 (58%) had normal QT intervals. In contrast, among the 34 patients not on diuretics, eight (24%) experienced QT prolongation and 26 (76%) had normal QT intervals. This difference was statistically significant (p = 0.005, χ² = 10.56 for diuretic users; p = 0.02, χ² = 5.91 for non-users), demonstrating that diuretic use is associated with a higher likelihood of QT interval prolongation in chronic liver disease patients (Table 5).

**Table 5:** Association Between Diuretic Use and QT Prolongation/Arrhythmias

Medication Use (Diuretics)	QT Prolongation (n = 59)	No QT Prolongation (n = 96)	p-value	Chi-square value
Diuretics Use (n=121)	51 (42%)	70 (58%)	0.005	10.56
No Diuretics Use (n=34)	8 (24%)	26 (76%)	0.02	5.91

Mortality was higher in patients with both QT prolongation and arrhythmias, with 10% (n = 5) of these patients dying during the study period, compared to 3% (n = 4) of patients with either QT prolongation or arrhythmias alone. Mortality was also higher in patients with cirrhosis (7%, n = 5) compared to non-cirrhotic patients (4%, n = 4) (Table 6).

**Table 6:** Mortality and Prognosis

Mortality Group	Value (n = 155)
Overall Mortality	9 (6%)
Mortality by Arrhythmia and QT Prolongation Status	
Both Prolonged QT and Arrhythmias	5 (10%)
Only Prolonged QT or Arrhythmias	4 (3%)
Mortality Rate by Cirrhosis	
Cirrhosis (Child-Pugh Score ≥7)	7% (5)
Non-cirrhotic (Child-Pugh Score <7)	4% (4)

## Discussion

This study aimed to explore the prevalence of QT interval prolongation and arrhythmias in patients with chronic liver disease (CLD) and to assess the underlying mechanisms contributing to these cardiac complications. The results prove that there is a strong correlation between the severity of liver diseases, QT prolongation, and arrhythmias. In particular, the cirrhotic patient population with severe impairments of the liver had a more substantial prevalence of prolonged QT and arrhythmias moments when compared to non-cirrhotic liver disease patients. In this research, the frequency of QT interval prolongation was 38% overall, with patients with cirrhosis being much more likely to experience QT prolongation (45%) than patients with non-cirrhotic liver disorder (19%). This observation is also in agreement with other studies, which have also found a prevalence of QT prolongation among patients with liver cirrhosis. Among the reasons, it is possible to mention the changed metabolism in cirrhosis, which influences the pharmacokinetics of drugs typically used by cirrhotic patients, e.g., diuretics and beta-blockers. In addition to that, cirrhosis is commonly conjoined by electrolyte abnormalities, especially hypokalemia and hypomagnesemia, which are both notoriously known to induce de elongation of the QT interval [15].

Electrolyte disturbances were significantly more frequent in patients with prolonged QT intervals, with hypokalemia present in 66% and hypomagnesemia in 54% of this group. These findings reinforce the established link between electrolyte imbalance and QT prolongation in chronic liver disease [16]. The prevalence of electrolyte imbalance among the patients of the CLD highlights the importance of the close observation of these parameters, particularly in individuals with severe liver pathology. In the study, 45% of the people demonstrated arrhythmias, of which atrial fibrillations were the most prevalent, and then came ventricular arrhythmias. Arrhythmias were significantly higher in the population of patients with cirrhosis (55%) than in those without (32%). These results are in line with earlier evidence claiming that the incidence of arrhythmias has been made higher in cirrhotic patients, especially atrial fibrillation and ventricular tachycardia [17]. This is especially alarming, since arrhythmias and long QT intervals may result in such serious complications as sudden cardiac death which is hard to predict in patients with liver disease. This study found very strong associations between the use of diuretics, a common prescription for CLD patients to treat ascites and edema, and diuretics with QT prolongation and heart arrhythmia [18,19]. Seventy-eight percent of the participants were taking diuretics, and 55% of those on diuretics had a QTc interval greater than 450 ms. This is important since diuretics can complicate the electrolyte imbalances, especially hypokalemia and hypomagnesemia, which will lead to QT prolongation [20]. In addition to that, the administration of diuretics to cirrhotic patients with already reduced renal function would appear to cause further consequences, like volume loss and the development of renal dysfunction that would exacerbate cardiac functioning. As a result, the electrolytes and renal functions, especially in the case of advanced liver disease, need to be closely monitored when diuretics are prescribed to such patients. Prolongation of QT and arrhythmias are often unappreciated adverse effects that occur frequently in this patient group [21]. The results indicate that patients with severe liver dysfunction and with cirrhosis merit close observation for QT prolongation and arrhythmias when they are taking medicine that can influence the work of the heart, including diuretics. Routine ECG monitoring and regular electrolyte assessment can be used to detect patients who are potentially at risk of developing arrhythmic events and allow the patient to be timely intervened to prevent the threat of developing life-threatening arrhythmia [22,23].

Limitations and future research

While this study provides valuable insights into the relationship between QT prolongation, arrhythmias, and chronic liver disease, there are several limitations. First, the study design is cross-sectional, meaning that causal relationships cannot be established. Longitudinal studies are needed to assess the long-term risks of arrhythmias and QT prolongation in CLD patients. Second, the sample size was relatively small, and the study was conducted at a single center, which may limit the generalizability of the findings. Future studies with larger, multi-center cohorts are needed to confirm the prevalence and risk factors for QT prolongation and arrhythmias in CLD patients across diverse populations.

## Conclusions

It is concluded that QT interval prolongation and arrhythmias are common complications in patients with CLD, particularly in those with cirrhosis and advanced liver dysfunction. The study demonstrated a significant association between liver disease severity and the occurrence of QT prolongation and arrhythmias, with electrolyte imbalances, particularly hypokalemia and hypomagnesemia, contributing to these cardiac abnormalities. Additionally, the use of diuretics, commonly prescribed in CLD patients, was found to exacerbate electrolyte disturbances, increasing the risk of both QT prolongation and arrhythmic events. The findings highlight the need for careful monitoring of cardiac function, including routine electrocardiograms (ECGs) and regular electrolyte assessments, in patients with chronic liver disease, especially those with cirrhosis.
